# Enhanced Nanoparticle Recognition via Deep Learning-Accelerated Plasmonic Sensing

**DOI:** 10.3390/bios14080363

**Published:** 2024-07-26

**Authors:** Ke-Xin Jin, Jia Shen, Yi-Jing Wang, Yu Yang, Shuo-Hui Cao

**Affiliations:** Department of Electronic Science, Xiamen University, Xiamen 361005, China

**Keywords:** surface plasmon microscopy, deep learning, nanoparticle sensing

## Abstract

Surface plasmon microscopy proves to be a potent tool for capturing interferometric scattering imaging data of individual particles at both micro and nanoscales, offering considerable potential for label-free analysis of bio-particles and bio-molecules such as exosomes, viruses, and bacteria. However, the manual analysis of acquired images remains a challenge, particularly when dealing with dense samples or strong background noise, common in practical measurements. Manual analysis is not only prone to errors but is also time-consuming, especially when handling a large volume of experimental images. Currently, automated methods for sensing and analysis of such data are lacking. In this paper, we develop an accelerated approach for surface plasmon microscopy imaging of individual particles based on combining the interference scattering model of single particle and deep learning processing. We create hybrid datasets by combining the theoretical simulation of particle images with the actual measurements. Subsequently, we construct a neural network utilizing the EfficientNet architecture. Our results demonstrate the effectiveness of this novel deep learning technique in classifying interferometric scattering images and identifying multiple particles under noisy conditions. This advancement paves the way for practical bio-applications through efficient automated particle analysis.

## 1. Introduction

**Figure 1:**
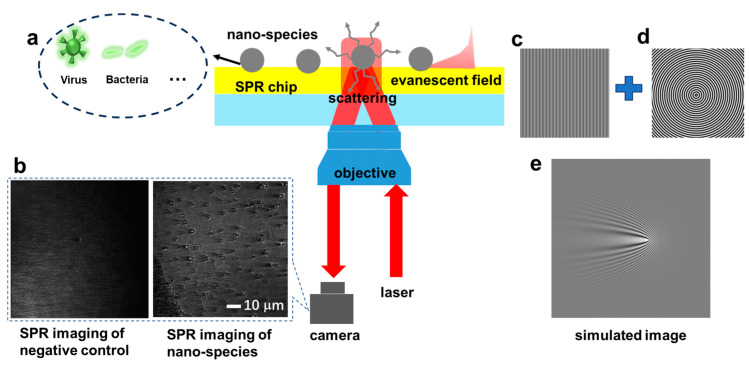
(**a**) The scheme of SPR image configuration to sense nano-species. Inset shows the typical SPR image of nano-species with wave-like patterns and parabolic tails to induce poor spatial resolution. (**b**) Images of the negative control and nano-species. (**c**) The simulated image of the plane wave. (**d**) The simulated image of the scattering wave. (**e**) The sum of images (**c**,**d**) resulting in the wave-like pattern observed in the simulated SPR image of a nano-species.

Surface plasmon (SP) is an electromagnetic surface wave that is formed due to the inhomogeneous distribution of free electrons on the surface of a metal. It refers to the electron density wave propagating on the metal surface formed by the interaction between free electrons and incident light. Surface plasmon resonance (SPR) occurs when the incident light interacts with the surface plasmon (SP) wave to absorb most of the light energy and then convert it into heat energy, resulting in almost no reflected light on the metal surface [1]. Based on SPR, surface plasmon microscopy (SPM) that can acquire resonance images has been developed to detect molecular interactions on metal surfaces [2]. One of the most noteworthy aspects of SPM technology is that it can achieve label-free detection to study interactions under the nanoscale, in which a single particle could scatter the SP wave on the metal surface excited by the incident light, inducing a parabolic shape pattern in the image (see Figure 1a). We obtain the SPR image of the film with a drop of PBS buffer as the negative control (Figure 1b). SPM has been applied in label-free imaging of biological nano-species [3,4], such as viruses [5], bacteria [6,7], and extracellular vesicles [8], etc.

Previous reports have illustrated that an SPM image should be formed by two basic processes, including the scattering of an SP wave by the object and interference of the scattered field with the propagating SP wave [9]. As shown in Figure 1c–e, the sum of a plane wave (Figure 1c) and a scattered plasmonic wave (Figure 1d) indeed result in a characteristic parabolic tail with the wave-like pattern to reduce the lateral resolution (Figure 1e). Processing SPM images in Fourier space has been used in image reconstruction for the classification of particle numbers, which enhances the high-frequency component and attenuates the low-frequency component to improve the contrast of the image, and then deconvolution is performed to obtain the reconstructed SPM image with significantly higher spatial resolution [10]. In this way, the number of scatterers can be predicted by finding intensity peaks in the reconstructed image. Although the intensity peaks of the reconstructed image are noticeable, the shape of the peaks would be distorted by neighborhood peaks. Once the two scatterers become closer, the two apparently separate peaks corresponding to the scatterers’ positions merge into a slightly visible but distorted peak. The reconstructed image will be completely distorted, hindering the correct prediction of scatterer number.

The development of artificial intelligence methods, especially deep learning [11,12,13], is gaining momentum. This progress is enabling parameter estimation of SPM images for fast identification, facilitated by advancements in image processing [14,15,16,17] and speech recognition [18]. For example, Moon et al. have used a convolutional neural network (CNN) to identify the number of particles based on a direct SPM image [19]. By comparing them with the image reconstruction method, they concluded that deep learning significantly improves the accuracy by about six times and is useful for the scattering estimation of polydisperse mixtures. Zhang et al. proposed that pre-trained neural network approach could be combined with multiangle illumination for detecting the number densities of nanoparticles [20]. Yu et al. integrated union network structures for digitally counting nanoparticle binding events [21]. Liu et al. employed VecNet for identifying nanoparticle composition using the phase parameter, but still needed manual identification mode to click the central point of the nanoparticles [22]. These studies indicate that achieving better recognition requires precise alignment of parameters within the physical model of particles, necessitating strict experimental conditions and complicating model establishment. Moreover, if the actual scatterer sizes are not uniform, recognition may fail. Real microscope images, even those obtained from simple test samples like silica particles, are subject to a variety of issues such as scattering, diffusion, and inconsistent illumination. These factors introduce significant variability and noise that are not fully captured in simulated datasets. Consequently, relying solely on simulated datasets is insufficient to train the network effectively, as it fails to adapt to the complexities and nuances of real experimental conditions. To achieve robust performance, it is essential to incorporate real-world data into the training process, ensuring the network can handle the diverse challenges presented by actual microscope images.

In this study, our aim is to leverage deep learning-based methods to analyze multi-particle representations. We adapt a theoretical model based on single-particle surface plasmon resonance (SPR) imaging with actual captured particle images to create a hybrid dataset to a network model for classifying and recognizing images with different numbers of scatterers. Deep learning technology has been shown to significantly improve classification accuracy, thereby enhancing the automation of SPR imaging measurements.

## 2. Experiments and Discussion

### 2.1. Experimental Sample Collection

In this paper, silica particles with a diameter of 150 nm, which were purchased from Bangs Laboratories, Inc. (150 nm, Catalog Number: NT06N), were used to simulate typical biological nanoparticles (e.g., virus, which have a similar size of about 200 nm and a consistent refractive index of 1.5 comparable to silica particles [23]) for SPM experimental measurements. The nanoparticles were dispersed in PBS buffer solution (pH 7.4), prepared with the recipe containing 137 mM NaCl, 2.7 mM KCl, 10 mM Na_2_HPO_4_, and 1.8 mM KH_2_PO_4_ and configured to 1.75 × 10^−3^ mg/mL. One drop was dried on a gold film cover slide (the thickness of the gold film was 50 nm) for imaging. Gold film cover chip was fabricated by a thermal evaporator (Auto306, Edwards, Crawley, England) with 2 nm chromium and 50 nm gold on the cleaned BK7 cover slips. One drop PBS without nanoparticles was used as the control sample. The SPM image was captured using a Kretschmann structure (Nikon Ti-U, 100×, 1.49 NA, Tokyo, Japan) coupled with an objective lens. Using a 100 × oil-immersion objective, the typical FOV is 90 µm × 85 µm with the optical diffraction limit resolution. As shown in the scheme of SPR image setup in Figure 1, nano-species surrounded by the plasmonic wave induce a scattering component that interferes with the plasmonic wave and generates a parabolic shape pattern in the SPR image. The excitation light was modulated as p-polarized light, the camera frame rate was 5 fps, and the exposure time was 1s. The incident light is reflected by the film beam splitter and converges on the focal plane of the objective lens. SPR is excited by parallel light irradiation at the gold–air interface and interacts with the nanoparticles at the interface to change the field distribution. Then, the far-field is reversely coupled to be collected by the objective lens, and then transmitted to the camera through the dichroic mirror and tube lens, and the SPM images of the nanoparticles are collected for analysis.

### 2.2. Selection of Deep Learning Networks

In deep learning, Convolutional Neural Network (CNN) is a class of deep neural networks that plays an important role in natural language processing, speech recognition, and computer vision. It is often used to extract features from images. With excellent hardware resources, better training results can often be achieved by scaling the convolutional network model, which requires balancing network depth, width, and input image resolution. Sandler et al. proposed MobileNetV2 based on the inverted residual structure, which effectively reduces the computational complexity and size of the network [24]. In 2019, Tan and Le investigated the influence of model width, depth, and image resolution on the network performance, determining the optimal ratio of the three parameters using neural architecture search [25]. They proposed a series of efficient models with high classification accuracy but fewer model parameters, named EfficientNet. As shown in Figure 1 of their article [25], EfficientNet outperforms other commonly used networks. The study [25] also demonstrates that EfficientNet performs better than other models on various datasets, such as ImageNet (ImageNet: A large-scale dataset with over 14 million images categorized into 20,000 different classes) (97.1%), CIFAR-100 (CIFAR-100: Stands for the “Canadian Institute For Advanced Research dataset,” which contains 100 different classes of images) (91.7%), and Flower (Flower: Refers to the “Oxford 102 Flower Dataset,” which consists of 102 categories of flowers commonly found in the United Kingdom) (98.8%), improving accuracy while speeding up the training and inference process.

EfficientNet comprises several networks, from EfficientNet-B0 to efficientnet-B7. EfficientNet-B0 is the most basic network structure. Compared to EfficientNet-B0, other EfficientNet variants target different image sizes and gradually increase the number of sub-blocks, channels, depth, and width, leading to more complex parameters and higher computational consumption. EfficientNet-B0, on the other hand, matches the size of the images in our dataset, has a relatively small number of parameters, and better meets our experimental needs. Therefore, we choose EfficientNet-B0 as our deep learning network.

**Figure 2:**
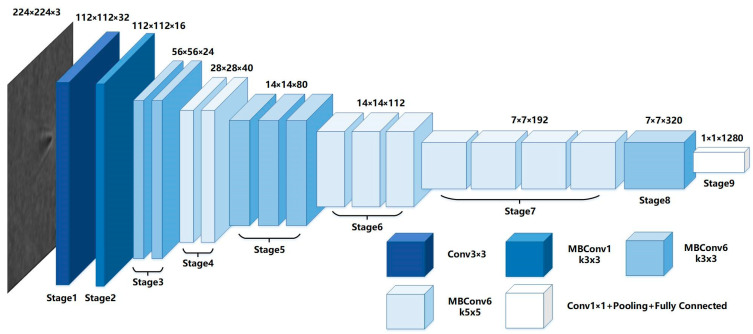
Schematic diagram of the EfficientNet-B0 architecture. It consists of the convolution layer (Conv), batch normalization (BN), rectified linear unit (Relu), max pooling layer (Pooling), fully connected layer (FC), and output layer.

The configuration of the EfficientNet-B0 model is depicted in Figure 2, which is formed by stacking nine stages. The first stage is a 3 × 3 convolutional layer, the second to eighth stages are stacked Mobile Inverted Bottleneck Convolution (MBConv) layers, and the last stage consists of a 1 × 1 convolutional layer, an average pooling layer, and a fully connected layer. The main block of EfficientNet is MBConv, first proposed and used in the MobileNetV2 network by Sandler et al. in 2018. During the stacking of the MBConv blocks, the model alternates between 3 × 3 and 5 × 5 convolutional layers to achieve better feature extraction. The input of the EfficientNet-B0 model is 224 × 224 RGB three-channel images, and then the resolution is continuously reduced to decrease the size of the feature map while the network width is expanded to increase the model learning accuracy. With the neural architecture search technique, EfficientNet-B0 obtains the optimal structure, which leads to a considerable breakthrough in the accuracy and speed of feature extraction. In this paper, the network is fine-tuned so that it can solve the problem more effectively, being able to identify particles and estimate their numbers.

### 2.3. Image Classification Experiments Based on the Simulated Image Dataset

#### 2.3.1. Preparation of Simulated Image Datasets

A general issue restricting the application of deep learning is the scarcity of data. It is well known that deep learning networks contain a large number of model parameters. Without the support of a large training set, it is easy to obtain overfitting, resulting in low accuracy of the trained network on the test set and the inability to be applied in practice. However, the uncontrolled distribution of nanoparticles and the challenge of highly sensitive measurement make it impractical to obtain sufficient high-quality experimental SPM images with ideal labels. Because this process involves changing the state, position, and experimental conditions of different particles and conducting corresponding experiments, it is very time-consuming and labor-intensive, which is difficult to put into practical experimental study.

Consequently, we generated simulation images through MATLAB based on an SP scattering physical model [9]. The SP scattering from a single nanoparticle can be described as a decaying spherical wave. The wave-like patterns observed in SPRM imaging of nanoparticles result from the interference between the evanescent wave of the incident field and the scattered field of the nanoparticle. By changing various parameters of the model to change the wavelength of the incident light, particle size, etc., we obtained sufficient samples to construct the dataset. This method is the most concise and convenient solution to obtain a variety of SPM images. By setting a certain range of random positions for the particles, the interference between the scatterers can also be obtained when multiple scatterers exist.

**Figure 3:**
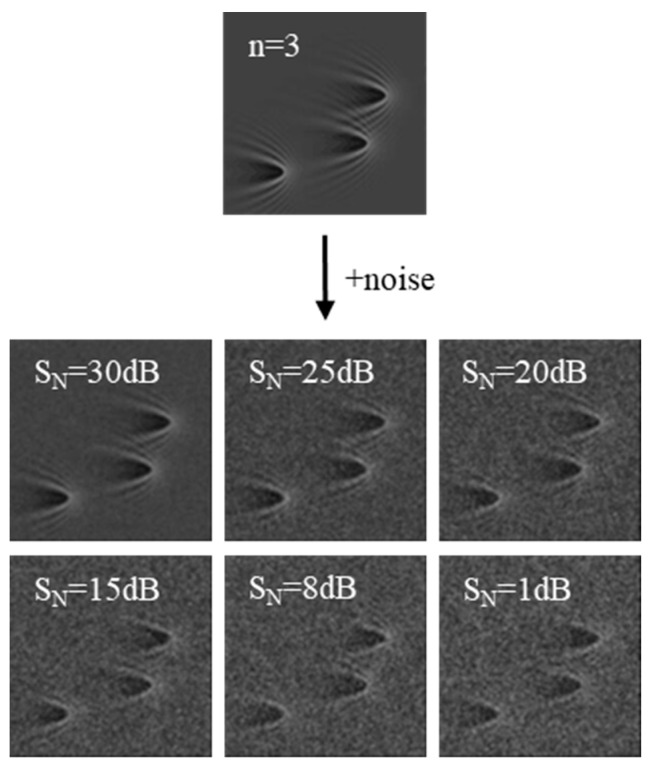
Simulated images with different signal-to-noise ratios based on mathematical model.

The simplified physical model based on the ideal state only considers the interference scattering of surface plasmon waves by nanoparticles. However, the image obtained in the experiment also contains the influence of optical systems, such as the objective lens and the unsatisfactory experimental environment, which leads to noise and other influencing factors. For example, the intensity and angle of incidence of light were not uniform over the sample surface, which made the SP scattering pattern of individual scatterers inhomogeneous. In addition, an SP image often contains motion artifacts caused by vibration, which make the background difficult to remove and result in low contrast of the image. Therefore, to enable the network to recognize the experimental image better, we need to mimic the degradation process on the basis of the ideal simulation image to simulate the experimental image. We added Gaussian noise and applied the blurring operation to generate the degraded image appropriately. Specifically, we used a disk-shaped filter for the blurring operation. The filter was created using the fspecial(‘disk’, 1) function in MATLAB, which generates a disk filter with a radius of 1. This filter was then applied to the image using the imfilter function with the ‘replicate’ boundary option. This method ensures that the image degradation is realistically simulated, which is crucial for understanding the potential failures and robustness of the model during the network training process. In Figure 3, the noise-free simulated image and those under noise conditions with different signal-to-noise ratios are shown. SN represents the signal-to-noise ratio of the image, which intuitively indicates the influence of noise on the quality of the simulated image.

#### 2.3.2. Experiments and Discussion Based on the Simulated Image Dataset

A simple parameter setting of the SPM physical model mentioned above allows us to generate particle images that are visually similar to real experimental ones. We assume that zero to nine particles are in the region of interest. Because the region can be split into subregions, the approach could also be generalized to the analysis of a large number of particles. Using MATLAB R2021b, zero to ten particles are generated with different levels of Gaussian noise. There are 7000 images for each particle number. The image data are randomly divided into the training set, the validation set, and the test set in the ratio of 8:1:1. By feeding this large amount of simulated data into the network, the deep learning algorithm continuously updates the network parameters through backpropagation, ultimately obtaining a trained network model with optimal parameters. By loading this trained model, we can directly recognize other simulated data or real data. All experiments were carried out on a computer server equipped with a 12th Gen Intel(R) Core(TM) i7-12700H (2.3 GHz, 14 cores per CPU), 32 GB RAM, and an RTX 4090 card. The proposed deep learning (DL) network was implemented in the open-source deep learning platform—Pytorch1.11.0.

**Figure 4:**
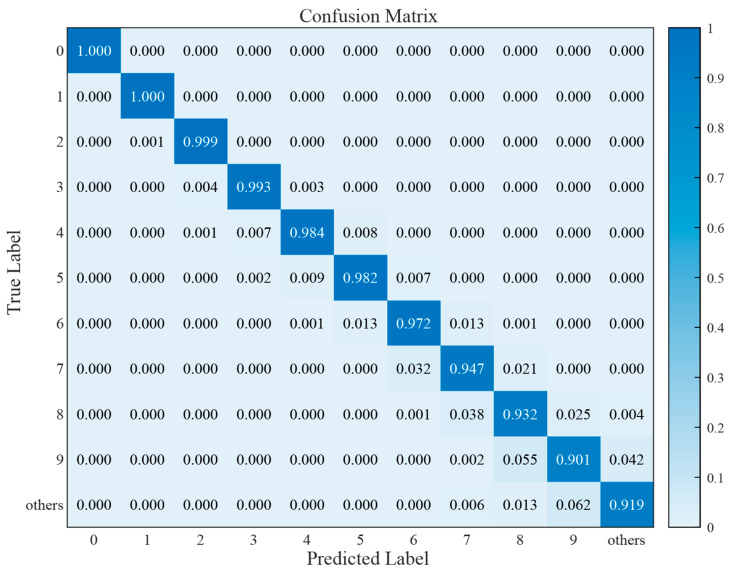
Confusion matrix representing the accuracy of classification. The left axis represents the actual number of particles in the picture, and the bottom axis represents the number of particles in the picture predicted by the network. The diagonal line from the top left to the bottom right represents the probability of correct predictions.

The network predicts a total of 11 categories: N = 0–9 and an ‘others’ category for all situations including N > 9. Generally, the accuracy of deep learning networks decreases as the number of particles increases, which is reasonable since distinguishing more crowded images (with a larger number of particles) is usually more challenging. The confusion matrix shown in Figure 4 aligns with this intuition. As shown in the figure, the particle recognition accuracy is 100% when N = 0 and 90.1% when N = 9, indicating a decreasing trend. For predictions in each category, there are two types of misidentifications: first, when the predicted number of particles is slightly more than the actual number; second, when the predicted number of particles is slightly less than the actual number. The only exception is the ‘other’ category, where all cases with N > 9 could be included. Here, there is only one type of misidentification: predicting fewer particles than the actual number. As a result, the accuracy of this category might be slightly higher than N = 9.

It also explains why we include an ‘others’ category as a prediction result. Without an ‘others’ category, the network fails to discriminate between N = 9 and N > 9, misidentifying both cases as N = 9, which might cause an artificial increase in the accuracy. In this case, the network is flawed and does not consider all possible cases when determining the number of particles. Retaining the ‘others’ category allows images that would not have been recognized correctly to be classified as ‘others’, thereby improving the recognition accuracy to better match real-world situations.

**Figure 5:**
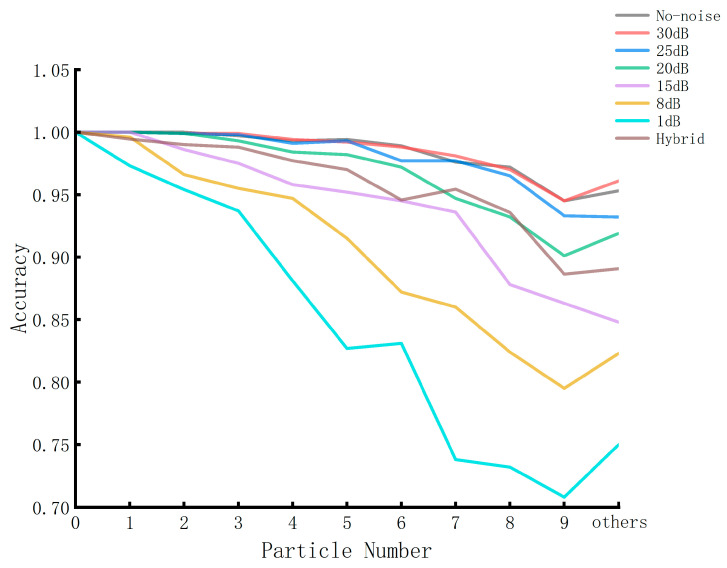
Accuracy curves versus different particle numbers with different signal-to-noise ratios. The curves of different colors illustrate how accuracy changes with particle number under varying noise levels and the hybrid noise conditions.

Subsequently, 1000 images were extracted from each category of simulation data with no noise and signal-to-noise ratios (SNRs) of 30 dB, 25 dB, 20 dB, 15 dB, 8 dB, and 1 dB to form a mixed dataset simulating the simultaneous presence of different noises. We trained the mixed dataset and plotted the results, comparing the accuracy obtained using the single-noise dataset mentioned earlier, as illustrated in Figure 5. Through the comprehensive observation of Figure 5, we can observe that the accuracy of the recognition results is close to 100% in the cases of no-noise, SN = 30 dB, and SN = 25 dB, indicating that the EfficientNet model has a strong recognition ability for simulated images in noise-free or low-noise cases. There is a decrease in recognition accuracy as the noise increases since the decrease in signal-to-noise ratio would increase the difficulty of identifying particles in the generated image. Moreover, as the number of particles increases, the mutual interference between particles grows and the peak distortion caused by the neighborhood particles also increases the difficulty of recognition, so the accuracy of recognition decreases when the number of particles grows. Furthermore, within the ‘others’ category, we observed a sudden rise in accuracy, affirming our previous conjecture. As the primary focus of this paper revolves around N = 0–9 particles, any misidentification within the ‘others’ category does not impact our conclusions. Overall, our deep learning network can provide a good recognition of particle numbers, whether in the case of multiple scattered particles or in the case of different levels of noise together. If the experimental noise is low enough, the application of deep learning in particle recognition and classification has great prospects for development and is worthy of research promotion.

### 2.4. Classification Experiments of Actual Images

#### 2.4.1. Dataset Description

**Figure 6:**
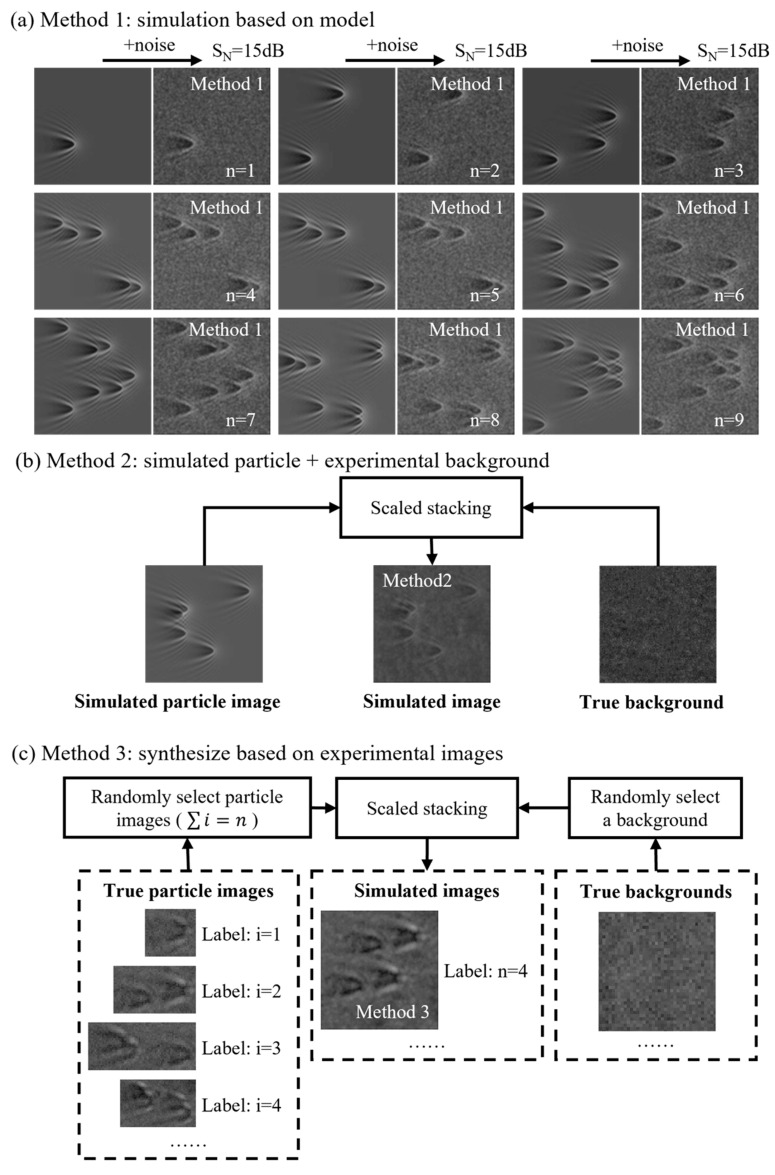
Composition of the hybrid dataset. (**a**) Method 1: simulation based on model; (**b**) Method 2: simulated particle + experimental background; (**c**) Method 3: synthesize based on experimental images.

Because the simulated images generated by mathematical simulations still differ from the actual images generated by real experiments, it is difficult to obtain sufficiently good performance if the network trained only on the simulated images is applied directly to the actual image classification task. In order to adopt our network in real-world situations, we made a hybrid dataset using data augmentation. In the hybrid dataset, there are 8100 images for each particle number. As shown in Figure 6, the hybrid dataset contains three categories of image data.

The first type of data are the simulation dataset generated according to Section 2.3.1. For each number of particles, we used the SP scattering model established in MATLAB to generate 1000 images randomly with seven different noise conditions: no noise and SNR of 30 dB, 25 dB, 20 dB, 15 dB, 8 dB, and 1 dB; 7000 images in total. In Figure 6a, we give the specific steps of image generation when SNR = 15dB. For the second type of data, we cut 300 background images without any particles from the real experimental images, which can better express the background noise caused by the interference of the optical system such as the objective lens and the unsatisfactory experimental environment. They were randomly used as backgrounds to add to the simulated particles; in this way 1000 images of each number of particles were generated, as shown in Figure 6b. For the third type of data, some particles were randomly intercepted from the acquired experimental images, and they were combined and arranged into 100 images each for N= 0–10.

Method 3 entails capturing particle images from experimental images to construct a particle image library, where each image is labeled with the corresponding particle count. Concurrently, background images are captured to build a background image library. To create a simulation image with a specific particle count, several images are selected from the particle image library corresponding to that count. Background images are then randomly selected from the background image library and superimposed onto them at random positions. This approach effectively expands the dataset of simulated images resembling actual experimental images.

#### 2.4.2. Experiments and Discussion Based on the Augmented Dataset

We first use the network, which has been trained on the dataset containing only the data generated with Method 1, to identify the actual particles. But this experiment did not yield the expected results. The main reason is that the acquired experimental images contain highly complicated backgrounds, including not only Gaussian noise but also speckles, due to the uneven plane of light incidence. The particle sizes in one image are also slightly different. Because of these factors, Method 1 (i.e., complete mathematical simulation) cannot be well adapted to the actual image under poor experimental conditions. Therefore, we needed to consider the practical situation and incorporate information from real experimental images. By complementing the mathematical simulation data with Methods 2 and 3, we obtained an augmented dataset that more closely resembled the experimental images, significantly improving the network’s performance.

**Figure 7:**
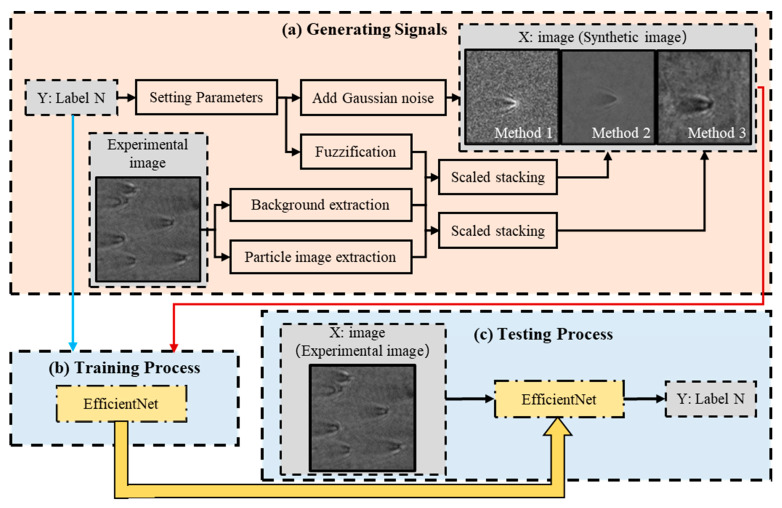
Flowchart to show (**a**) signal generation, (**b**) training processing, and (**c**) testing processing in automated SPM analysis.

The overall experimental process is shown in Figure 7. First, we trained a feasible deep learning model to learn the relationship between the SP scattering parameters of light and the number of scatterers in the experimental image. This process, shown in Figure 7b,c, includes two parts: training and testing. Since deep learning networks require large amounts of data for training, and collecting and labeling sufficient experimental data is challenging, we designed a more convenient dataset generation scheme. We established the SP particle scattering model using MATLAB, set reasonable parameters such as particle wavelength size, and generated a large amount of simulation data by adding different levels of noise. Additionally, we collected some real experimental data to ensure accurate results. These data were further processed using the three methods detailed in Section 2.4.1 to produce a mixed dataset for subsequent use. By combining a large amount of simulated data with a smaller amount of real experimental data, we generated a training dataset that provided sufficient data for effective network training. The resulting network could determine the number of scatterers with high accuracy, as shown in Figure 7a.

**Figure 8:**
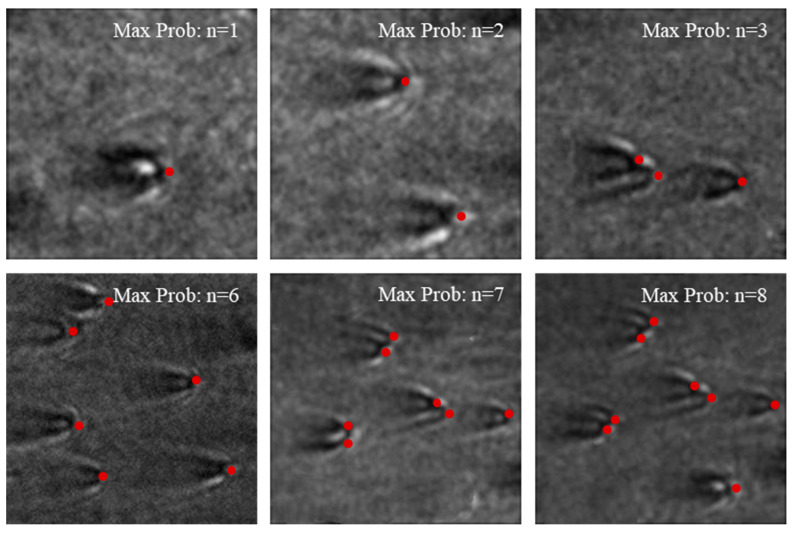
Estimation results of particle number on experimental images. The red dots indicate the distinguished particles by the network in the pictures.

Figure 8 shows the results when the number of particles is correctly estimated by the network trained on the augmented dataset. We used real particle images obtained in the experiment to test the trained network and selected part of the results to showcase. The red dots in the figures denote the peak positions of each particle. The results demonstrate that the modified dataset and network perform well in particle recognition. Even when there are three, seven, and eight particles in the image with certain degrees of overlap, the deep learning method outperforms manual classification or classic image reconstruction methods, reflecting its superiority and feasibility. We also measured the time taken by the network to identify the number of particles, which averaged only 32.3 ms. Compared to manual identification, the network significantly reduces the time in identifying the number of particles, thereby accelerating the analysis of surface plasmon microscopy.

## 3. Conclusions

In this paper, we have proposed an automated analysis method for SPM images that combines interference scattering models with deep learning techniques. While it is conventional to support deep learning methods with simulated data, the disparity between simulated and experimental data and the lack of sufficient experimental data for training limit their application to real-world problems. This challenge is particularly evident in particle identification, where simulated data alone provide limited assistance in achieving accurate experimental results.

We overcame this obstacle by creating a hybrid dataset that combines a large amount of simulated data with a small amount of experimental data, thereby improving recognition accuracy. By training a neural network with an EfficientNet structure on this hybrid dataset, we achieved high accuracy in particle recognition and identification, even under noisy conditions with multiple particles.

This experiment serves as an initial exploration of the feasibility of applying deep learning to particle recognition. Several related issues merit further investigation. For instance, using deep learning to generate images that closely resemble experimental ones through techniques like Generative Adversarial Networks (GANs) could be highly beneficial. Additionally, designing a network to make actual images more similar to simulated ones, such as through appropriate denoising of particle images, is a promising avenue. These efforts are currently underway, and we believe that more advanced deep learning-assisted techniques will significantly enhance their practical applications in biosensing.

## Data Availability

The datasets generated during and analyzed during the current study are not publicly available, but are available from the corresponding author on reasonable request.
